# Whole-genome sequencing and evolutionary analysis of the wild edible mushroom, *Morchella eohespera*

**DOI:** 10.3389/fmicb.2023.1309703

**Published:** 2024-02-01

**Authors:** Yixin Li, Ting Yang, Jinxia Qiao, Jian Liang, Zhonghu Li, Wei Sa, Qianhan Shang

**Affiliations:** ^1^State Key Laboratory of Plateau Ecology and Agriculture, Academy of Agriculture and Forestry Sciences, Qinghai University, Xining, China; ^2^Key Laboratory of Resource Biology and Biotechnology in Western China (Ministry of Education), College of Life Sciences, Northwest University, Xi’an, China

**Keywords:** *Morchella eohespera*, genome evolution, phylogeny, positive selection analysis, gene expansion, gene contraction

## Abstract

Morels (*Morchella*, Ascomycota) are an extremely desired group of edible mushrooms with worldwide distribution. *Morchella eohespera* is a typical black morel species, belonging to the Elata clade of *Morchella* species. The biological and genetic studies of this mushroom are rare, largely hindering the studies of molecular breeding and evolutionary aspects. In this study, we performed *de novo* sequencing and assembly of the *M. eohespera* strain m200 genome using the third-generation nanopore sequencing platform. The whole-genome size of *M. eohespera* was 53.81 Mb with a contig N50 of 1.93 Mb, and the GC content was 47.70%. A total of 9,189 protein-coding genes were annotated. Molecular dating showed that *M. eohespera* differentiated from its relative *M. conica* at ~19.03 Mya (million years ago) in Burdigalian. Evolutionary analysis showed that 657 gene families were contracted and 244 gene families expanded in *M. eohespera* versus the related morel species. The non-coding RNA prediction results showed that there were 336 tRNAs, 76 rRNAs, and 45 snRNAs in the *M. eohespera* genome. Interestingly, there was a high degree of repetition (20.93%) in the *M. eohespera* genome, and the sizes of long interspersed nuclear elements, short interspersed nuclear elements, and long terminal repeats were 0.83 Mb, 0.009 Mb, and 4.56 Mb, respectively. Additionally, selection pressure analysis identified that a total of 492 genes in the *M. eohespera* genome have undergone signatures of positive selection. The results of this study provide new insights into the genome evolution of *M. eohespera* and lay the foundation for in-depth research into the molecular biology of the genus *Morchella* in the future.

## 1 Introduction

*Morchella* is a member of the Morchellaceae family in the Pezizales order of the Pezizomycetes class (Du, 2019). The genus *Morchella* species has a beauteous cap with a honeycomb-like structure and a brown, yellow, black, or pale color that looks similar to open lamb tripe, giving it the name “morels.” According to the color and shape characteristics of the fruit body, the genus Morels can be divided into four major groups: black, yellow, red, and half-open morels (Bunyard et al., 1995) The results of recent molecular systematic studies showed that Morels can be divided into three branches, namely Esculenta Clade, Elata Clade (including two groups of black and half-open morel), and Rufobrunnea Clade support (Bunyard et al., 1995; Guzmán and Tapia, 1998). Among them, the Esculenta Clade and Elata Clade branches are sister groups and constitute the main group of the Morel genus (Min et al., 2017). There are currently 32 species identified in the Elata Clade branch (Du, 2019). True morels (*Morchella*) are supposed to have evolved in the early Cretaceous in the northern hemisphere, where they now show a high degree of continental endemicity (Murat et al., 2018). Biogeographical studies have shown that the species of the Elata Clade branch were mainly distributed in Europe, North America, South America, and Asia (Du et al., 2012; Du, 2019). There are at least 16 species of black morels in China (Du, 2019). Some species, such as *Mel*-14 (Sichuan), *Mel*-33 (Gansu), and *Mel*-34 (Yunnan), showed regional geographic distribution characteristics. It is reported that *Morchella* has a high edible value because it contains enough basic amino acids, vitamins, mineral elements, and proteins (Irfan et al., 2017; He et al., 2018). Furthermore, the *Morchella* species has important medicinal values for its multiple pharmacological effects, including anticarcinogenic (Hu et al., 2013), antioxidant (Cai et al., 2018; Li et al., 2018), and immunomodulatory activities (Su et al., 2013; Yang et al., 2019).

Genomics research is a window to understanding a concrete species. Some species in the genus *Morchella* were sequenced and analyzed. The genome study of *Morchella septimelata* M. Kuo is the first example of the *Morchella* genome, the size of which was 49.81 Mb (Li et al., 2018). The genome sequence deepened our understanding of the mechanisms of secondary metabolite biosynthesis and provided some insights into the growth, development, and carbohydrate degradation of this species. Subsequently, the genome sequence of another cultivated species, *Morchella sextelata* M. Kuo, has also been published (Mei-Han et al., 2019). The genome of *M. sextelata* is larger than that of *M. septimelata*, with a size of 52.93 Mb. The *M. sextelata* genome facilitates the study of gene components, protein-coding genes, annotated biological functions, and secondary metabolite gene clusters. Two different polar monospore strains of *Morchella importuna*, M. Kuo, O’Donnell, and T.J. Volk, were used for genomics research (Masaphy, 2010; Liu et al., 2018), further expanding our understanding of morel biology and evolution and facilitating the molecular genetic analysis and breeding of *M. importuna*.

In recent years, the cultivation of several *Morchella* species has been successfully commercialized in China. However, *Morchella eohespera* Beug, Voitk, and O’Donnell (*Mel*-19), as a wild morel, is still harvested from the wild at sites distributed in Qinghai, Xinjiang, Yunnan, and Gansu Provinces, as well as in other places in China. There is no cultivation record or genomic analysis of this species (Du et al., 2017). *M. eohespera* is a typical black morel species with a black pileus, honeycomb-like surface, conical to widely conical, and a white hollow stalk. The main habitats are moist, sandy, calcareous soil, or calcareous bedrock under grass or trees (Voitk et al., 2016).

To investigate the genetic organization and provide data for further studies of the biological functions of *M. eohespera*, a *de novo* whole-genome sequence analysis was conducted, and the genome was assembled. Additionally, the protein-coding genes, gene components, and related biological functions were analyzed. At the same time, a comparative study was carried out with the genomes of other closely related fungi, aiming to provide genomic data for further research on the evolutionary aspects and biological functions of *M. eohespera*.

## 2 Materials and methods

### 2.1 Strain selection and molecular identification

In this study, the fruiting body of wild *M. eohespera* strain m200 was collected from Makehe Forest Farm in Qinghai Province, China (E 100°86′70″, N32°69′74″). The mycelium of the m200 strain was cultured on potato dextrose agar (PDA) medium for 2 weeks at a temperature of 25 ± 1°C. The Ezup column Fungal Genomic DNA Extraction Kit (Sangon Biotech, Shanghai, China) was used to extract genomic DNA from the m200 strain. The integrity of the DNA was assessed by electrophoresis on a 0.7% (w/v) agarose gel. The quality of the extracted genomic DNA was determined from the A_280_/A_260_ ratio using a NanoDrop One spectrophotometer (NanoDrop Technologies, Wilmington, DE, United States) and a Qubit 3.0 fluorometer (Life Technologies, Carlsbad, CA, United States). The genes for the m200 strain’s rRNA internal transcribed spacer (ITS), translation elongation factor 1-alpha (*ef1-α*), RNA polymerase II subunit 1 (*rpb1*), and RNA polymerase II (*rpb2*) were amplified and sequenced to aid species identification by comparing this sequence with known fungal sequences in the NCBI GenBank database with BLASTX (Supplementary Table S1). DNA sequences were aligned, and species were identified. Molecular Evolutionary Genetic Analysis (MEGA) version 7.0 was used for species evolutionary distance analysis (Wattam et al., 2014; Kumar et al., 2016; Wattam et al., 2017).

### 2.2 Genome sequencing and assembly

The genome of the m200 strain was sequenced using the third-generation Nanopore Sequencing Technology on the Oxford Nanopore platforms at Goalgene (Wuhan, China) (Lu et al., 2016). A library comprising >1 kb fragments met the requirements for sequencing. Finally, after the sequencing data of Nanopore were obtained, the high-quality nanopore reads were corrected and assembled using Canu v1.5 (Koren et al., 2017) software. The minimap 2 2.17 (Li, 2018) comparison method and racon v1.3.1 (Chen et al., 2020) error correction method were used to paste the original third-generation off-machine data back to the assembled genome for error correction analysis. The software purge haplotigs (Roach et al., 2018) were used to de-redundant the genome after initial assembly error correction and identify and remove redundant heterozygous contigs based on the depth distribution of reads and sequence similarity.

The genome is compared with the second- and third-generation data in the NCBI nucleotide (NT) database. Additionally, the completeness of the genome was assessed using BUSCO v5.1.2 (Benchmarking Universal Single-Copy Orthologs) with fungi_odb10 (Simão et al., 2015).

### 2.3 Genomic prediction and genome annotation

#### 2.3.1 Repeat sequence prediction and annotation

After obtaining the whole-genome data of the m200 strain, transposon sequence analysis was carried out for the assembled gene sequences with the transposon Repbase database (Bao et al., 2015), using RepeatMasker (Tarailo-Graovac and Chen, 2009) and RepeatProteinMasker software. Meanwhile, based on its own sequence ratio [Software: RepeatModeler (Zeng et al., 2018)] and repeat sequence characteristics [Software: Trf (Benson, 1999) and LTR-FINDER (Ou and Jiang, 2019)] were used for *de novo* prediction (Saha et al., 2008). Default parameters were used.

#### 2.3.2 Gene prediction and function annotation

Two strategies were used for gene prediction: (1) based on *Ab initio* gene prediction, with GlimmerM (Majoros et al., 2003) and Augustus v 3.3.1 (Nachtweide and Stanke, 2019) software, the gene model was predicted *ab initio* and (2) based on homology-based prediction (Hamp et al., 2013), where we selected five closely related species (*Ascobolus immersus* Pers, *Choiromyces venosus* (Fr.) Th. Fr., *Sphaerosporella brunnea* (Alb. & Schwein.) Svrček & Kubička, *Terfezia boudieri* Chatin, and *Tuber magnatum* Picco) to predict the genomic genes of the M200 strain. Then, with the help of MAKER2 (Campbell et al., 2014) software, we integrated the gene sets predicted by the two methods into a non-redundant and more complete gene set. Additionally, the results of CEGMA v2.5 was also integrated (Parra et al., 2007).

Several complementary methods were used to annotate the assembled sequences. The genes were annotated by aligning the sequence with those previously stored in different protein databases including the Gene Ontology (GO) (Ashburner et al., 2000), Kyoto Encyclopedia of Genes and Genomes (KEGG) (Kanehisa et al., 2006), Nr (Non-Redundant Protein Database) (Yu and Zhang, 2013), Swiss-Prot (Magrane and Consortium, 2011), TrEMBL (O’Donovan et al., 2002), and KOG (Eukaryotic Orthologous Groups) (Tatusov et al., 2003). Transcription factors were annotated according to their InterPro IDs in the Fungal Transcription Factor Database (Wilson et al., 2008).

#### 2.3.3 Non-coding RNA annotation

The tRNA sequences in the genome were identified using the tRNAscan-SE software (Chan and Lowe, 2019). Since rRNA is highly conserved, we chose the rRNA sequence of a closely related species as the reference sequence and utilized BLASTN (Rivas and Eddy, 2001) comparison to search for rRNA in the genome. Rfam (Kalvari et al., 2018) predicted other non-coding RNAs, such as microRNA (miRNAs) and small nuclear RNAs (snRNAs).

### 2.4 Gene family construction

The sequences of proteins ≥30 aa (amino acids) of *M. eohespera* and 14 other fungi were employed to compute pairwise similarities using BLASTP 2.7.1 (Altschul et al., 1990) (E-value ≤10^−5^). Using the OrthoMCL v2.0.9 pipeline with an inflation value of 2.0, gene families were constructed. Default parameters were used.

### 2.5 Phylogeny reconstruction and divergence time estimation

The m200 strain was analyzed with 13 Ascomycota *Morchella conica* Pers. (Lütkenhaus et al., 2019), *Morchella crassipes* (Vent.) Per. (Liu et al., 2020), *Morchella eximia* Boud. (Liu et al., 2020), *M. importuna* (Du et al., 2017), *M. septimelata* (Liu et al., 2018), *M. sextelata* (Masaphy, 2010), *Ascodesmis nigricans* Tiegh. (Liu et al., 2020), *Beauveria brongniartii* (Sacc.) Petch (Shang et al., 2016), *Neurospora crassa* Shear & B.O. Dodge (Baker et al., 2015), *Parastagonospora nodorum* (Berk.) Quaedvl., Verkley & Crous (McDonald et al., 2019), *Rhynchosporium agropyri* Zaffarano, B.A. McDonald & A. Linde (Penselin et al., 2016), *Tuber melanosporum* Vittad. (Martin et al., 2010), and *Aspergillus niger* Tiegh. (Gebru et al., 2020). One Basidiomycota [*Gloeophyllum trabeum* (Pers.) Murrill (Floudas et al., 2012)] was added to root the phylogenetic trees. Based on orthoMCL clustering, single-copy ortholog gene groups from 15 fungal species were selected randomly and aligned separately using MUSCLE v3.8.31.^1^ Gblocks were used to identify and remove poorly aligned regions.^2^ Then, maximum-likelihood tree estimation and bootstrap analyses were performed with RAxML v8.0.24 (Stamatakis, 2015) (m: GTRGAMMA). The maximum-likelihood (ML) analysis uses the default settings, and statistical support values were obtained through 100 replicates using non-parametric bootstrapping.

According to the ML tree, the species differentiation time provided by the TimeTree database^3^ was referred to as the fossil time (Kumar et al., 2017), and the BEAST v1.8.0 (Drummond and Rambaut, 2007) software was used to estimate the differentiation time of these eight species. We applied a general time reversible (GTR) model for nucleotide substitution and the “Yule process” tree prior model with three calibration points. The divergence time was estimated by Markov Chain Monte Carlo (MCMC) analysis for 80,000,000 generations. Based on fossil calibrations at the two calibrated nodes, including the divergence time of *Morchella* and the Tuberaceae, black morels (Elata clade) and yellow morels (Esculenta clade) (O’Donnell et al., 2011). According to the molecular clock theory, this study used the coding sequence (CDS) alignment of 1,220 single-copy gene family sequences to estimate the differentiation time (Liu et al., 2019). The orthologous genes of *T. melanosporum* were used as the outgroup.

### 2.6 Analysis of expansion and contraction of gene families and positive selection gene analysis

Using the cluster analysis results from gene families, the CAFE v4.2.1 software (Lu et al., 2017) was employed to examine gene family expansion and contraction with a significance level of 0.05.

To detect whether a gene family is affected by positive selection, the PAML software package’s CODEML PAML4.9/CODEML (model 4, kappa 0, codon2, blen 0) tool was utilized for each gene family (Yang, 2007).

### 2.7 Synteny analysis

The software minimap2 2.17 was utilized for conducting pairwise genome comparisons (Li, 2018) and for visualizing the comparison outcomes. We initially created an index, subsequently compared it, and ultimately obtained the comparison result in the same format. After comparing the results, it was determined that the R package ‘pafr’ was best suited for visualization purposes, and a collinear point diagram was consequently drawn.

### 2.8 Large fragment copy analysis and genome-wide replication

The lastz 1.04.00^4^ software (Gao and Miller, 2020) developed by rsharris/lastz was utilized to search for the syntenic segments within the genome and to compare the repetitive fragments contained within it with the statistics of the genome.

Two analytical methods were chosen for genome-wide replication. One is synteny analysis (4DTV, Fourfold Degenerate Synonymous Site Synteny), while the other is grounded on the Ks distribution map (Huang et al., 2009). The MCscanX software was utilized to search for gene pairs in the syntenic region of the genome for synteny analysis (Wang et al., 2012), followed by MUSCLE for gene comparison, which eventually calculated its 4DTV value and generated a distribution map. Another approach involved identifying gene pairs within the genome through homologous clustering. MUSCLE was employed to perform gene comparison, calculate the Ks value, and generate a distribution map.

## 3 Results

### 3.1 Species identification

We identified the fungal species by analyzing the sequences of four nuclear gene fragments of the m200 DNA: ITS, *ef1-α*, *rpb1*, and *rpb2*. Species identification was performed by comparing the sequence with the sequence of known fungi in the NCBI GenBank. Finally, combined with the morphological analysis, the *Morchella* strain m200 was confirmed as *M. eohespera*.

### 3.2 Molecular sequencing and *de novo* assembly

In this study, whole-genome sequencing was performed for *M. eohespera*, based on third-generation nanopore sequencing technology. After filtering out the low-quality reads, a total of 64.19 Gb of Oxford Nanopore long reads was obtained (Supplementary Table S2). The largest read length was 191,615 bp. The average read length is 12,668.38 bp, and the N50 read length is 27,302 bp.

**Figure 1:**
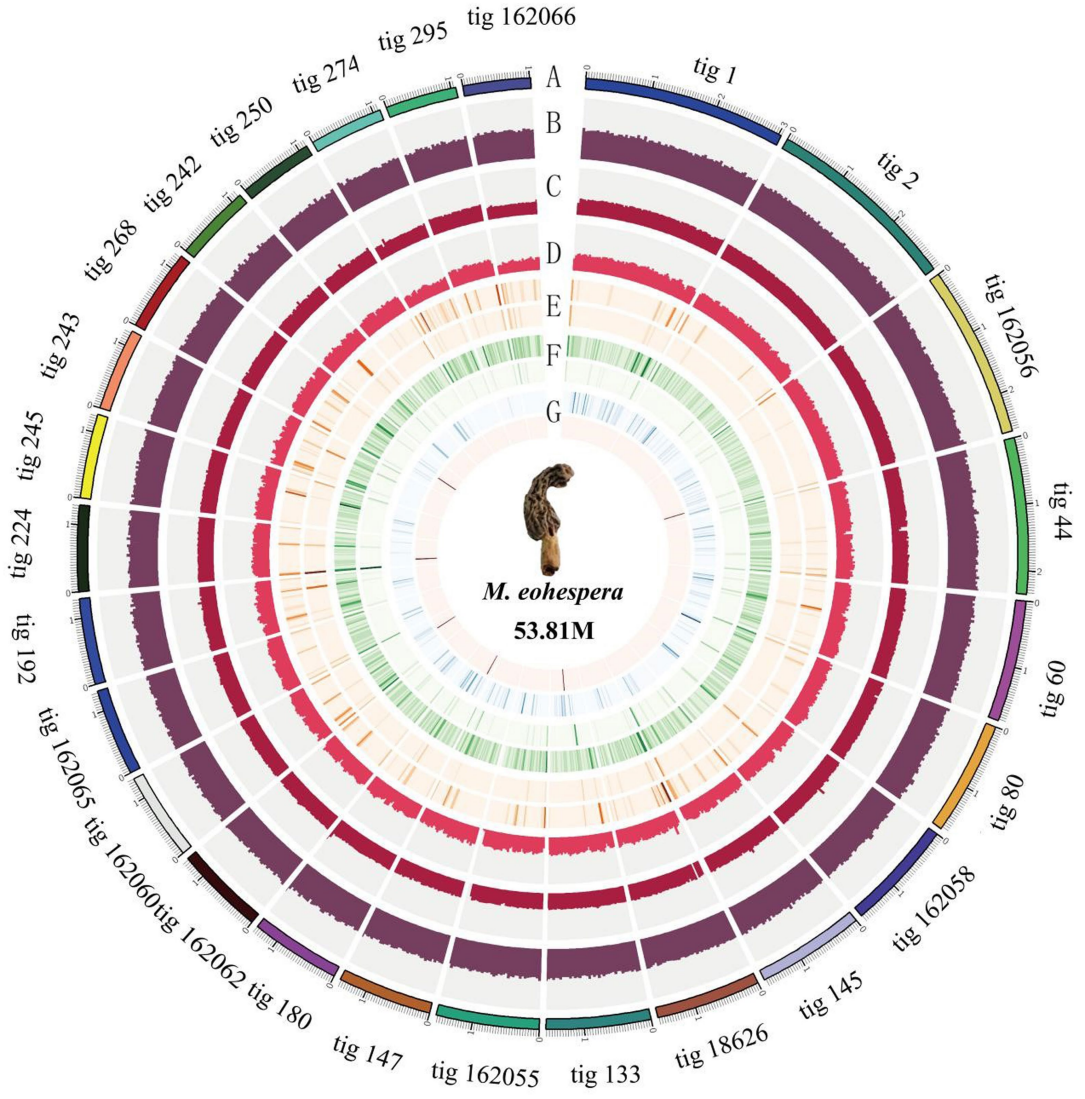
*Morchella eohespera* whole-genome map. From outside to inside, in order: **(A)** genomic information; **(B)** GC content distribution; **(C)** second-generation reads depth distribution; **(D)** depth distribution of three generations of reads; **(E)** outer circle is a homozygous SNP distribution, and the inner circle is a heterozygous SNP distribution; **(F)** outer circle is a homozygous InDel distribution, and the inner circle is a heterozygous InDel distribution; and **(G)** complete comparison of BUSCO gene distribution on the genome: blue is single-copy BUSCO and red is duplicated BUSCO.

Due to the unavailability of reference information about the genome of *Morchella*, a *de novo* assembly strategy was used to assemble the *M. eohespera* genome. The result shows that the size of the assembled genome is approximately 59.66 Mb; after correction, it is approximately 53.81 Mb, and the GC content of the sample genome is approximately 47.70% (Table 1). In addition, based on the second-generation and third-generation data of the *M. eohespera* genome, we constructed a complete genome map of *M. eohespera* (Figure 1).

**Table 1:** *De novo* genomic assembly results of *Morchella eohespera*.

*Morchella eohespera*	Length/%
Total length (bp)	53,808,214
Max length (bp)	3,806,699
N50 (bp)	1,933,924
N90 (bp)	1,385,344
GC Content (%)	47.70

By comparing with the genome data of other species of *Morchella*, we obtained a high-quality genome sequence through the Nanopore sequencing platform, with a sequencing depth of 1,193× with the N50 length reaching 1.93 Mb (Table 2).

**Table 2:** Whole-genome assembly statistics of different species of *Morchella*.

Species	Strain	Assembly(Mb)	GC%	Scaffolds	Contigs	N50	Genome coverage	INSDC	Sequencing	Assembly method
*Morchella eohespera*	M200	53.81	47.70	31	31	1,933,924	1,193X	–	MinION Nanopore	*De novo* canu
*Morchella conica*	SH	52.4255	46.9	207	2,692	41,756	17.0X	VOVZ00000000.1	Illumina MiSeq	Newbler v. 2.8
*Morchella importuna*	M04M26	51.0782	47.3	106	110	958,716	298X	QOKS00000000.1	Illumina HiSeq	AllPaths v. 44,849
*Morchella sextelata*	NZTD180501373	52.9253	47.4	59	59	1,569,782	108.82X	SDUU00000000.1	PacBio	*De novo* SMRT Link v. 5.1.0
*Morchella crassipes*	M10	56.7561	47.2939	23	64	1,330,510	400.0x	WBVU00000000.1	Illumina HiSeq; PacBio RS	AllPaths v. 44,849; DBG2OLC v. 5.1; SSPACE v. 3.0
*Morchella eximia*	MG90	73.46	46.0	7,793	10,613	26,474	57x	QMFK00000000.1	Whole-genome shotgun (WGS)	Platanus version v. 1.2.1

### 3.3 Repeat sequence prediction annotation

In this study, *de novo* prediction and comparison of homologous sequences were used to annotate the repetitive sequences of *M. eohespera*. There are 11.26 Mb of repetitive sequences in *M. eohespera*, accounting for approximately 20.93% of the genome (Table 3). The type and content analysis results of transposable elements (TEs) in the genome of *M. eohespera* showed that almost all plant genome transposons exist in the genomes of *M. eohespera*, with long terminal repeats (LTRs) being the main type of TE. The *M. eohespera* genome contains approximately 4.56 Mb long terminal repeats, accounting for approximately 8.48% of the whole *M. eohespera* genome, indicating that the expansion of LTR may have caused the expansion of the genome of *M. eohespera* (Table 4).

**Table 3:** Statistics of repeat sequence.

Type	Repeat size (bp)	% of genome
Trf	1,662,266	3.09
Repeatmasker	1,588,249	2.95
Proteinmask	1,032,973	1.92
*De novo*	9,968,180	18.53
Total	11,261,609	20.93

(a) TRF is a tandem repeat sequence in the genome sequence found by TRF software; (b) RepeatMasker is a transposon element obtained by annotating genome sequence through RepeatMasker software based on RepBase library; (c) ProteinMask is a transposon element obtained by annotating genome sequence through RepeatProteinMask software based on RepBase library; (d) de novo is the result of using the final sequence file obtained by the software RepeatModeler and LTR-FINDER as a library, and annotating the genome sequence through the RepeatMasker software; and (e) total is the result obtained by the above various methods, and the non-redundant result after removing the overlap between them.

**Table 4:** Statistics of transposon type.

	RepBase TEs		TE Proteins		*De novo*		Combined TEs	
	Length (bp)	% of Genome	Length (bp)	% of Genome	Length (bp)	% of Genome	Length (bp)	% of Genome
DNA	363,684	0.68	60,223	0.11	1,021,052	1.90	1,365,765	2.54
LINE	187,364	0.35	2,487	0.00	662,625	1.23	829,583	1.54
SINE	1,112	0.00	0	0.00	8,395	0.02	9,060	0.02
LTR	966,800	1.80	970,263	1.80	4,394,194	8.17	4,563,034	8.48
Satellite	25,245	0.05	0	0.00	234,673	0.44	253,386	0.47
Simple_repeat	121,226	0.23	0	0.00	430,390	0.80	534,002	0.99
Other	66	0.00	0	0.00	0	0.00	66	0.00
Unknown	6,656	0.01	0	0.00	4,038,735	7.51	4,045,313	7.52
Total	1,588,249	2.95	1,032,973	1.92	9,303,117	17.29	9,918,004	18.43

(a) RepBase TEs and TE proteins are based on the transposon elements obtained by annotating the genome through the RepeatMasker and RepeatProteinMask software, respectively, based on the RepBase library; (b) de novo is the repetitive sequence obtained by repeatModeler and LTR-FINDER of de novo prediction method as the library, and the result of the repetitive sequence in the genome obtained by the software RepeatMasker; and (c) combined TEs is the result of integrating the above three methods and removing redundancy.

### 3.4 Gene prediction and function annotation

Finally, 9,189 genes were annotated in the genome. The average gene length of the predicted genes of *M. eohespera* is 1,822 bp, the average CDS length is 1,317 bp, and the average exon length is 402.77 bp (Supplementary Table S3).

The whole genome of *M. eohespera* was annotated using the InterPro, GO, KEGG_ALL, KEGG_KO, Swiss-Prot, TrEMBL, Pfam, Nr, and KOG databases. The total number of *M. eohespera* genes with predicted functions was found to be 7,825, accounting for 85.16% of the total number of *M. eohespera* genes through functional cluster analysis (Table 5). Among them, there were 4,266 GO-annotated genes, accounting for 46.43% of the total. KEGG annotated 7,335 genes, accounting for 79.82% of the total. The remaining 15% of the genes could not be found in the currently known databases and belong to the unique genes of *M. eohespera*. These genes are likely to play an important role in the growth of *M. eohespera*.

**Table 5:** Gene annotation results of *Morchella eohespera*.

Database	Number	Percent (%)
InterPro	6,013	65.44
GO	4,266	46.43
KEGG_ALL	7,335	79.82
KEGG_KO	3,474	37.81
Swiss-Prot	4,364	47.49
TrEMBL	7,640	83.14
Pfam	5,877	63.96
NR	7,642	83.16
KOG	4,368	47.54
Total	10,497	91.13

### 3.5 Non-coding RNA

Non-coding RNA (ncRNA) plays a vital role in biological processes. The non-coding RNA prediction results showed that a total of 336 tRNAs were predicted in the *M. eohespera* genome, accounting for 0.053% of the entire genome. Compared with the amount of tRNA, the numbers of rRNA and snRNA were much lower, only 76 and 45, respectively. However, miRNA and snRNA were not predicted (Supplementary Table S4). The total number of ncRNA was 457, representing 0.94% of the genome assembly; this suggested that ncRNA formed only a small proportion of the overall genome size.

### 3.6 Identification of specific gene families and specific genes of *Morchella Eohespera*

Based on the sequence similarity of genes, the orthologous and paralogous relationships of 15 fungal genomes (*M. eohespera*, *M. conica*, *M. crassipes*, *M. eximia*, *M. importuna*, *M. septimelata*, *M. sextelata*, *A. nigricans*, *B. brongniartii*, *N. crassa*, *P. nodorum*, *R. agropyri*, *T. melanosporum*, *A. niger*, and *G. trabeum*) gene families were constructed. A total of 9,189 genes of the predicted genes of *M. eohespera* were clustered into 7,996 families, of which 48 gene families were unique to *M. eohespera* (Supplementary Table S5).

### 3.7 Phylogenetic analysis and divergence time estimation

**Figure 2:**
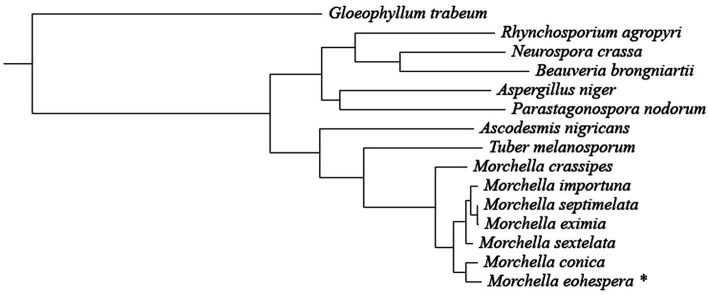
Phylogenetic tree constructed from seven *Morchella* species and eight related fungi. The taxon with * is the research object of this research.

A total of 1,220 single-copy orthologous genes were identified in the 15 fungal species (*Morchella eohespera*, *Morchella conica*, *Morchella crassipes*, *Morchella eximia*, *Morchella importuna*, *Morchella septimelata*, *Morchella sextelata*, *Ascodesmis nigricans*, *Beauveria brongniartii*, *Neurospora crassa*, *Parastagonospora nodorum*, *Gloeophyllum trabeum*, *Rhynchosporium agropyri*, *Aspergillus niger*, and *Tuber melanosporum*). A phylogenetic tree was constructed using the maximum-likelihood method and the GTRGAMMA model with 1,220 single-copy genes identified in the orthology analysis (Figure 2).

**Figure 3:**
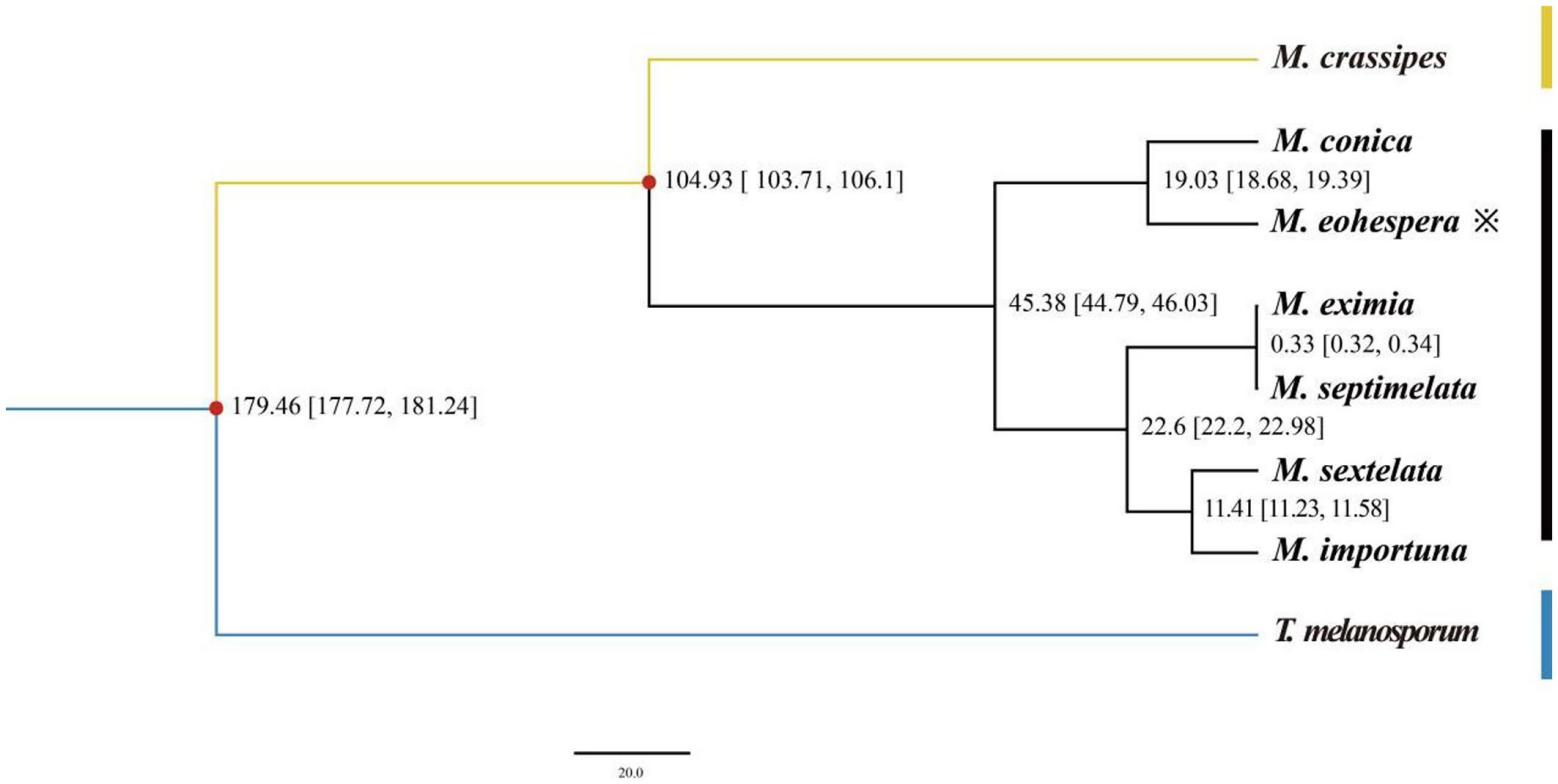
Evolutionary divergence time in eight species. The numbers on the branches indicate the estimated time of differentiation (million years ago, Mya), XXX-XXX differentiation time (X ~ X million years ago), and the red dots indicate fossil evidence.

It can be seen from Figure 2 that all *Morchella* species are clustered on a single evolutionary branch, with black morel and yellow morel (*M. crassipes*) being divided into two branches. Among them, *M. conica* was the closest relative to the *M. eohespera* species in one clade. *M. eohespera* is phylogenetically closest to *M. conica*, diverging ~19.03 million years ago (Figure 3).

### 3.8 Contraction and expansion of gene families

**Figure 4:**
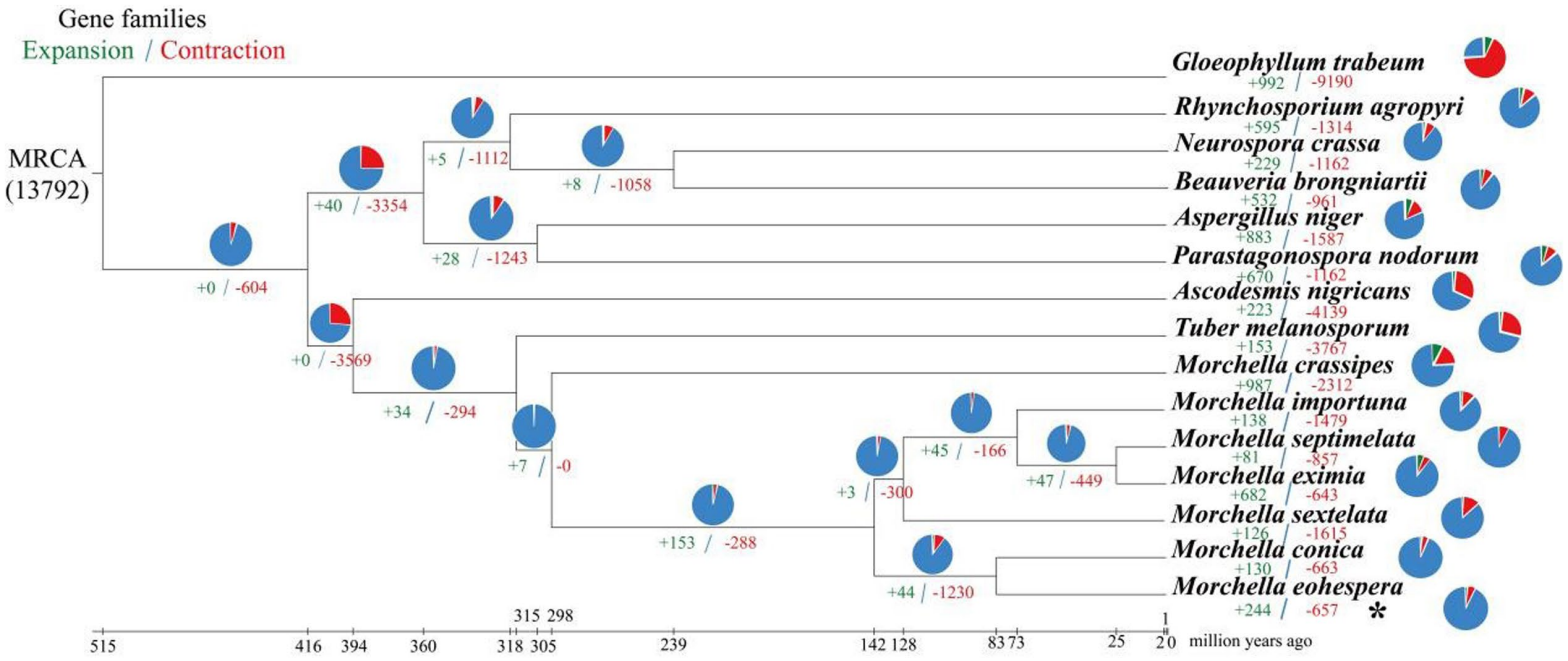
Expansion and contraction of *Morchella eohespera* gene families. The numbers on the branches of the phylogenetic tree indicate gene deletion (red) and gain (green). The pie chart to the right of each species name indicates the percentage of gene family amplification (green) and shrinkage (red) of that species. The pie chart to the right of the developmental tree shows the percentage of families that have changed (orange) and stayed the same (blue) among all species.

A phylogenetic tree was constructed using 1,220 single-copy genes from eight related fungi and seven species of *Morchella*. Among the 15 species, the gene families of *M. eximia* expanded more than contracted, whereas the other 14 species all showed more contraction than expansion (Figure 4). The number (657) of contraction gene families in *M. eohespera* is greater than the number (244) of expanded gene families, among which there are 244 expanded gene families and 657 contraction gene families. We performed an enrichment analysis on shrinkage genes (Supplementary Table S6). The contracted genes of *M. eohespera* are mostly involved in the “metabolic process” (GO:0008152), “cellular process” (GO:0009987), “organic substance metabolic process” (GO:0071704), and “primary metabolic process” (GO:0044238).

### 3.9 Enrichment analysis of positive selection genes

**Figure 5:**
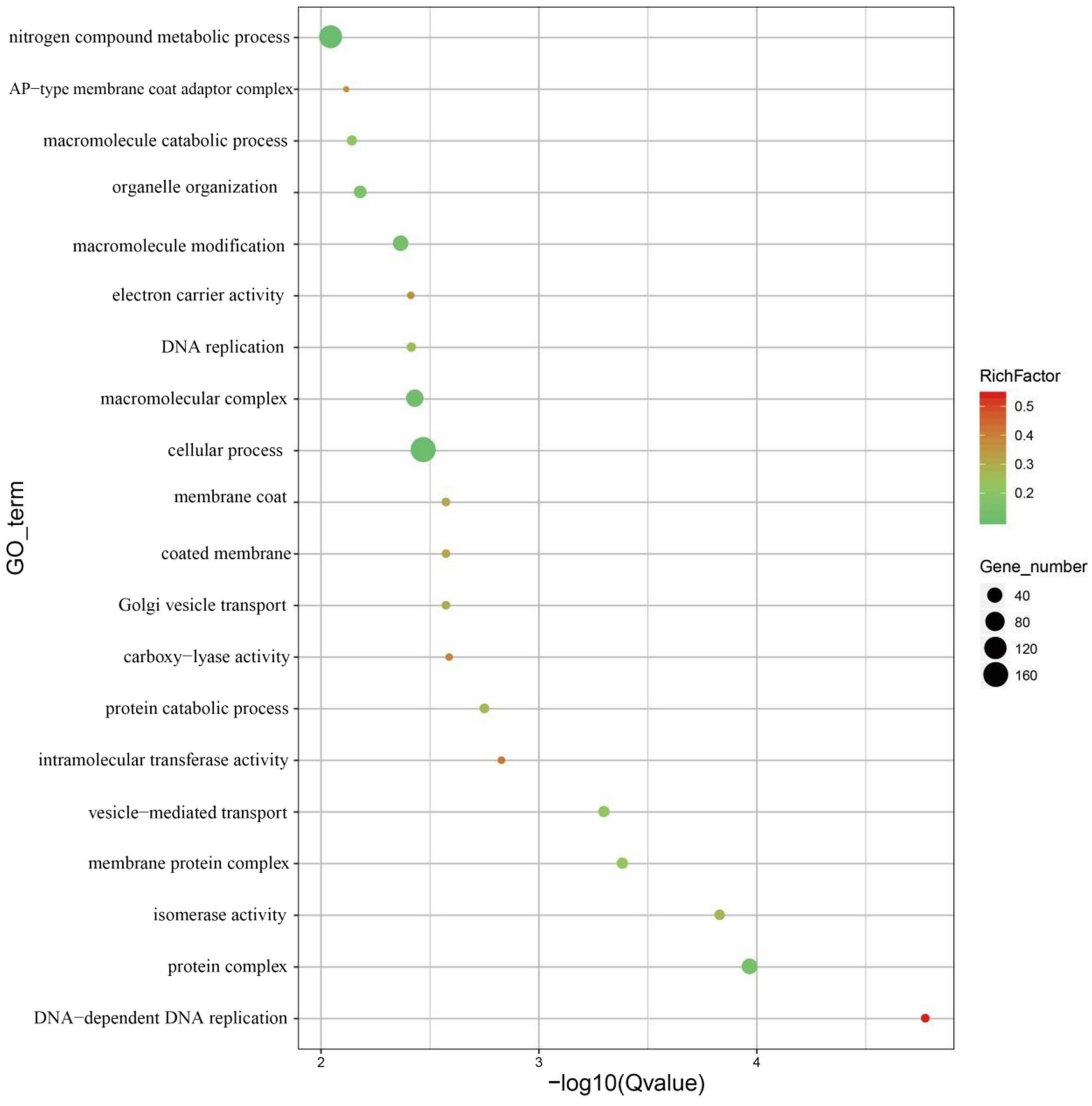
Display of GO enrichment results for positive selection genes. The abscissa is −log10 of the enriched *Q*-value, the ordinate is the GO term, the abscissa represents the number of genes in each category, and the ordinate represents the enriched genes.

The CODEML tool in the PAML software package was used to select a branch-site model to detect whether a certain gene family of *M. eohespera* was subject to positive selection. A total of 492 genes in the *M. eohespera* genome displayed signatures of positive selection (see Figure 5).

According to the GO database, genes subject to positive selection were mainly distributed in four functional entries: “Binding” (GO:0005488), “Catalytic activity” (GO:0003824), “Metabolic process” (GO:0008152), and “Cellular process” (GO:0009987) (Supplementary Figure S1). To better understand the gene functions in *M. eohespera*, we successfully assigned putative proteins to their orthologs in the KEGG database. The KEGG function classification is shown in Supplementary Figure S2. Analysis of the *M. eohespera* species-specific genes revealed that 15 genes were significantly enriched in various KEGG pathways, including “Proteasome” (ko03050), “Autophagy-animal” (ko04140), “mTOR signaling pathway” (ko04150), and “Cell cycle” (ko04110) (Supplementary Figure S3).

### 3.10 Gene synteny analysis

The synteny analysis was performed using minimap2 software. We selected two related species (*M. conica* and *M. sextelata*) of *M. eohespera* based on the phylogenetic tree and performed syntenic analysis (Supplementary Figures S4, S5). According to the results of the synteny analysis of the three *Mochella* species, we can infer that the synteny between *M. eohespera* and *M. conica* is high, which is consistent with the phylogenetic analysis results we constructed earlier, and the relationship between them is close (Supplementary Figure S6).

### 3.11 Large fragment copy analysis whole-genome replication

The lastz 1.04.00 software was used to count the number of repetitive fragment pairs contained in the synteny segment of the *M. eohespera* genome. The total number of *M. eohespera* SD fragments is 176, the median length is 2,403 bp, and the total length is 878,958 bp (Table 6).

**Table 6:** Large fragment replication statistics.

Cut off (bp)	Block	Median size (bp)	Genome coverage (bp)
>1 K	176	2,403	878,958
>5 K	45	7,697	681,925
>10 K	11	12,141	522,793
>50 K	3	54,947	334,771

We used two methods for detection, one being synteny analysis (4DTV distribution of gene pairs in the synteny region), and the other being based on the Ks distribution map (Ks distribution of best hit gene pairs on the whole genome). According to the 4DTV distribution map (Supplementary Figure S7) and the Ks distribution map (Supplementary Figure S8) of *M. eohespera*, combined with the statistical results of large fragment replication, it was found that no genome-wide replication occurred in the *M. eohespera* genome.

## 4 Discussion

The draft genome of *M. eohespera* (53.81 Mb) is slightly larger than that of the closely related species, *M. conica* and *M. sextelata*, which are 52.43 Mb and 52.93 Mb, respectively (Murat et al., 2018). The average gene length of *M. eohespera* (1,643 bp) is also slightly larger than that of *M. sextelata* (1,372 bp) and *M. septimelata* (1,571 bp). Furthermore, the GC content of the *M. eohespera* genome (47.70%) is also greater than that of *M. sextelata* (47.37%) and *M. septimelata* (47.40%) (Li et al., 2018; Liu et al., 2018). The *M. eohespera* genome was predicted to contain 699 complete BUSCO genes and 35 fragmented BUSCO genes, and the completeness of the genes was 92.2% (699/758) (Supplementary Table S7). Through the above comparison, we can see that the results of this study are true and credible. The differences in genome size, average gene length, and GC content among closely related species of *M. eohespera* are not very obvious. With the development of sequencing technology, our future research data will be more authentic. The results of this study provide sequence data resources for the molecular biology of *Morchella* fungi and lay the foundation for further research into improving this genus, which is characterized by its significance in medicine and gastronomy.

Repetitive DNA sequences are widely distributed in the genomes of eukaryotes, and repetitive sequences are closely related to the evolution, inheritance, and variation of species (Aguileta et al., 2008). The genome of *Morchella crassipes*, representing the first yellow morel genome published, was slightly larger than that of *M. eohespera*, but the proportion of the genome that represented repeat sequences in *M. eohespera* (20.93%) was clearly greater than that of *M. crassipes* (15.34%) (Liu et al., 2020). Transposons are of great significance in the study of species formation, biological evolution, gene expression regulation, and transgenic technology. The four most common types of transposons, namely DNA, long interspersed nuclear elements (LINEs), long terminal repeats (LTRs), short interspersed nuclear elements (SINEs), and a small number of other unknown types of transposons, were predicted in the *M. eohespera* genome.

Phylogenetic trees based on a single gene or several genes may produce inconsistent topological structures, whereas phylogenetic trees based on the series of available genes in the whole genome can provide relatively high resolution (Dooner and Weil, 2007). In the current study, we used 1,220 genome-wide single-copy orthologous protein-encoding sequences combined with data from 14 reference fungal species to construct the maximum-likelihood tree at the higher level of *M. eohespera*. The evolutionary tree showed that *M. eohespera* and *M. conica* were clustered into the smallest group, with synteny analysis by minimap2 showing a greater synteny between them.

The BEAST v1.8.0 software was used to estimate the differentiation time of *Morchella* species and related species (Figure 3). Based on the fossil calibration point, the divergence time of each species could also be calculated. Seven morel species were clustered in one branch, and two black morels, *M. eohespera* and *M. conica*, had the closest genetic relationship, with a differentiation time of approximately 19.03 Mya. A yellow morel species, *M. crassipes*, had a greater genetic distance from the six black morel species and differentiated at approximately 104.93 Mya. The Morchellaceae (seven morel species) were differentiated from the Tuberaceae (*T. melanosporum*) at the family level at approximately 179.46 Mya, a finding that was consistent with those from a previous study (Liu et al., 2020). On the contrary, the number of expanded gene families was greater than the number of contracted gene families in the *M. crassipes* genome in the previous study. The number of expanded and contracted genes in the current study of *M. crassipes* was 149 and 2,152, respectively, more than were reported in previous studies.

Gene family contraction and expansion analysis showed that *M. eohespera* and *M. crassipes* differed markedly in gene types. It was calculated that 987 genes expanded in *M. crassipes*, sharply more than the 743 genes in *M. eohespera* (Liu et al., 2020). On the other hand, 657 genes were contracted in *M. eohespera*, clearly less than 1,655 genes in *M. eohespera*. The number of contracted and expanded genes in *M. crassipes* was the largest of the seven *Morchella* species in this current study. Functional enrichment analysis reflected that the main function of the contracted genes of *M. eohespera* was related to the “metabolic process” (GO:0008152).

The genome sequencing in this study provides the first annotation of the whole-genome sequence of *M. eohespera*. This study may provide important data for evaluating the species of *Morchella*, improving culture techniques, and discovering bioactive compounds. This can help meet the increasing demand for *M. eohespera*, but it is also significant for ongoing research into *M. eohespera*. To provide additional information, the gene annotation file generated in this study was uploaded and may provide useful data in the future for further research on the differences between various *Morchella* species and their biological functions.

## 5 Conclusion

The importance of fungi in agriculture, human health, and ecology emphasizes their potential for biotechnological applications. Third-generation sequencing technology was used to sequence a high-quality *M. eohespera* genome. Using the relevant information from the *M. eohespera* genome, an accurate picture was generated of the phylogenetic relationship and evolution of *M. eohespera* and related species, providing a new reference genome for the evolutionary analysis of ascomycete fungi. The generation of the genome sequence of *M. eohespera* will help us to study the phylogenetic status of *M. eohespera* at the genome level and to mine the sequence for key candidate genes for valuable biological traits, laying a theoretical foundation for the artificial cultivation of *M. eohespera* for high-value food production and herbal medicines, and to conserve wild populations from extinction.

## Data availability statement

The original contributions presented in the study are publicly available. This data can be found here: https://www.ncbi.nlm.nih.gov/bioproject/; PRJNA1034038.

## Author contributions

YL: Writing – original draft, Data curation. TY: Software, Writing – original draft. JQ: Methodology, Writing – review & editing. JL: Formal analysis, Methodology, Writing – review & revision. ZL: Supervision, Writing – original draft. WS: Funding acquisition, Writing – original draft. QS: Formal analysis, Visualization, Writing – review & editing.

## Footnotes

^1^ http://www.drive5.com/muscle/

^2^ http://molevol.cmima.csic.es/castresana/Gblocks.html

^3^ http://www.timetree.org/home

^4^ http://www.bx.psu.edu/
