# Supplementary material for: Whole-genome sequencing and evolutionary analysis of the wild edible mushroom, Morchella eohespera
Source: Front Microbiol. 2024 Feb 1;14:1309703. doi: 10.3389/fmicb.2023.1309703 (PMC10868677; doi:10.3389/fmicb.2023.1309703)
Supplement: Supplementary file 1 [file Data_Sheet_1.docx]

**Table S1** PCR amplification primer sequence and annealing temperature information of *Morchella eohespera*

| **Primer name** | **sequence** | **Annealing temperature (℃)** |
| --- | --- | --- |
| ITS | ITS1: TCCGTAGGTGAACCTGCGG  ITS4: TCCTCCGCTTATTGATATGC | 55 |
| EF1-α | F: ACTCCTAAGTACTATGTCACCGTCATT  R: TGGAGAGGAAGACGGAGAGGCTT | 56 |
| RPB1 | F: TATATCACGTCGGTATGTATCCACTC  R: ATTTGCTCGGATGATCTCAG | 56 |
| RPB2B | F: TAGGTAGGTCCCAAGAACACC  R: GATACCATGGCGAACATTCTG | 57 |

**Table S2 Three-generation sequencing data statistics**

|  | **Total Base** | **Total Reads** | **Max Len** | **Avg Len** | **N50** | **L50** | **N90** | **L90** | **meanQ** |
| --- | --- | --- | --- | --- | --- | --- | --- | --- | --- |
| Raw data | 71,599,901,417 | 6,012,595 | 809,158 | 11,908.31 | 26,930 | 908,190 | 5,910 | 2,848,420 | 9.28 |
| Clean data | 64,199,462,911 | 5,067,693 | 191,615 | 12,668.38 | 27,302 | 806,876 | 6,225 | 2,491,692 | 10.01 |

**Table S3 Basic statistical results of gene prediction**

| Gene set | Protein coding gene number | Average gene length (bp) | Average CDS length (bp) | Average exon per gene | Average exon length (bp) | Average intron length (bp) |
| --- | --- | --- | --- | --- | --- | --- |
| denovo/AUGUSTUS | 8,485 | 1,643 | 1,338 | 3.90 | 343.13 | 105.24 |
| denovo/GlimmerHMM | 11,504 | 1,818 | 1,464 | 3.05 | 479.99 | 172.78 |
| homo/Ascobolus_immersus | 7,324 | 1,260 | 904.60 | 3.00 | 301.49 | 177.56 |
| homo/Choiromyces_venosus | 9,789 | 1,354 | 981.51 | 3.01 | 326.06 | 185.42 |
| homo/Sphaerosporella_brunnea | 8,799 | 1,446 | 978.98 | 3.05 | 321.28 | 228.14 |
| homo/Terfezia_boudieri | 8,300 | 1,228 | 879.53 | 2.96 | 297.09 | 177.72 |
| homo/Tuber_magnatum | 8,497 | 1,292 | 1,007 | 3.14 | 320.68 | 133.14 |
| BUSCO | 271 | 2,783 | 1,602 | 6.57 | 243.78 | 211.98 |
| MAKER | 7,630 | 1,966 | 1,392 | 3.85 | 418.61 | 123.74 |
| HiCESAP | 9,189 | 1,822 | 1,317 | 3.71 | 402.77 | 120.02 |

**Table S4 The Statistics of ncRNA**

| Type |  | Copy | Average length(bp) | Total length(bp) | % of genome |
| --- | --- | --- | --- | --- | --- |
| miRNA |  | 0 | 0 | 0 | 0 |
| tRNA |  | 336 | 85 | 28523 | 0.053009 |
| rRNA | rRNA | 76 | 198 | 15030 | 0.027933 |
|  | 18S | 4 | 1542 | 6168 | 0.011463 |
|  | 28S | 0 | 0 | 0 | 0 |
|  | 5.8S | 7 | 152 | 1064 | 0.001977 |
|  | 5S | 65 | 120 | 7798 | 0.014492 |
| snRNA | snRNA | 45 | 158 | 7107 | 0.013208 |
|  | CD-box | 23 | 141 | 3253 | 0.006046 |
|  | HACA-box | 3 | 205 | 615 | 0.001143 |
|  | splicing | 19 | 170 | 3239 | 0.00602 |
|  | scaRNA | 0 | 0 | 0 | 0 |

**Table S5 Gene family clustering results statistics**

| Species | Genes number | Genes in families | Unclustered genes | Family number | Unique families | Single copy genes | Average genes per family |
| --- | --- | --- | --- | --- | --- | --- | --- |
| *Morchella eohespera* | 9,189 | 7,996 | 1,193 | 7,315 | 48 | 1,220 | 1.093 |
| *Morchella conica* | 8,019 | 7,829 | 190 | 7,298 | 2 | 1,220 | 1.073 |
| *Morchella crassipes* | 8,976 | 8,548 | 428 | 7,118 | 58 | 1,220 | 1.201 |
| *Morchella eximia* | 10,332 | 9,520 | 812 | 7,664 | 422 | 1,220 | 1.242 |
| *Morchella importuna* | 8,051 | 7,816 | 235 | 7,247 | 5 | 1,220 | 1.079 |
| *Morchella septimelata* | 8,051 | 7,924 | 127 | 7,446 | 0 | 1,220 | 1.064 |
| *Morchella sextelata* | 8,032 | 7,820 | 212 | 7,266 | 6 | 1,220 | 1.076 |
| *Ascodesmis nigricans* | 9,154 | 6,081 | 3,073 | 5,599 | 77 | 1,220 | 1.086 |
| *Beauveria brongniartii* | 9,595 | 7,756 | 1,839 | 6,808 | 148 | 1,220 | 1.139 |
| *Neurospora crassa* | 9,726 | 7,133 | 2,593 | 6,632 | 85 | 1,220 | 1.076 |
| *Parastagonospora nodorum* | 15,966 | 8,661 | 7,305 | 7,510 | 241 | 1,220 | 1.153 |
| *Rhynchosporium agropyri* | 13,661 | 8,585 | 5,076 | 7,491 | 188 | 1,220 | 1.146 |
| *Tuber melanosporum* | 7,381 | 5,989 | 1,392 | 5,701 | 24 | 1,220 | 1.051 |
| *Aspergillus niger* | 10,422 | 8,656 | 1,766 | 7,093 | 194 | 1,220 | 1.22 |

Note: Unclustered genes refers to the number of genes unique to the species; Unique families refers to gene families unique to the species;

**Table S6 *Morchella eohespera* shrinkage gene GO enrichment (p<0.01)**

| GO term | Gene number | Backgroud gene number | RichFactor | Qvalue |
| --- | --- | --- | --- | --- |
| nucleobase-containing compound metabolic process | 8 | 598 | 0.0134 | 0.0e+00 |
| DNA metabolic process | 8 | 138 | 0.0580 | 0.0e+00 |
| cellular aromatic compound metabolic process | 8 | 649 | 0.0123 | 0.0e+00 |
| nitrogen compound metabolic process | 8 | 1233 | 0.0065 | 0.0e+00 |
| metabolic process | 8 | 1994 | 0.0040 | 0.0e+00 |
| cellular process | 8 | 1573 | 0.0051 | 0.0e+00 |
| DNA integration | 8 | 11 | 0.7273 | 0.0e+00 |
| cellular nitrogen compound metabolic process | 8 | 784 | 0.0102 | 0.0e+00 |
| macromolecule metabolic process | 8 | 1044 | 0.0077 | 0.0e+00 |
| cellular metabolic process | 8 | 1293 | 0.0062 | 0.0e+00 |
| primary metabolic process | 8 | 1430 | 0.0056 | 0.0e+00 |
| cellular macromolecule metabolic process | 8 | 960 | 0.0083 | 0.0e+00 |
| heterocycle metabolic process | 8 | 641 | 0.0125 | 0.0e+00 |
| organic substance metabolic process | 8 | 1517 | 0.0053 | 0.0e+00 |

Table S7 BUSCO evaluation

| Type | fungi_odb10 | | eukaryota_odb10 | |
| --- | --- | --- | --- | --- |
| Complete BUSCOs | 699 | 92.2 | 240 | 94.1 |
| Complete Single-Copy BUSCOs | 690 | 91.0 | 238 | 93.3 |
| Complete Duplicated BUSCOs | 9 | 1.2 | 2 | 0.8 |
| Fragmented BUSCOs | 35 | 4.6 | 12 | 4.7 |
| Missing BUSCOs | 24 | 3.2 | 3 | 1.2 |
| Total BUSCO groups searched | 758 | 100.0 | 255 | 100.0 |


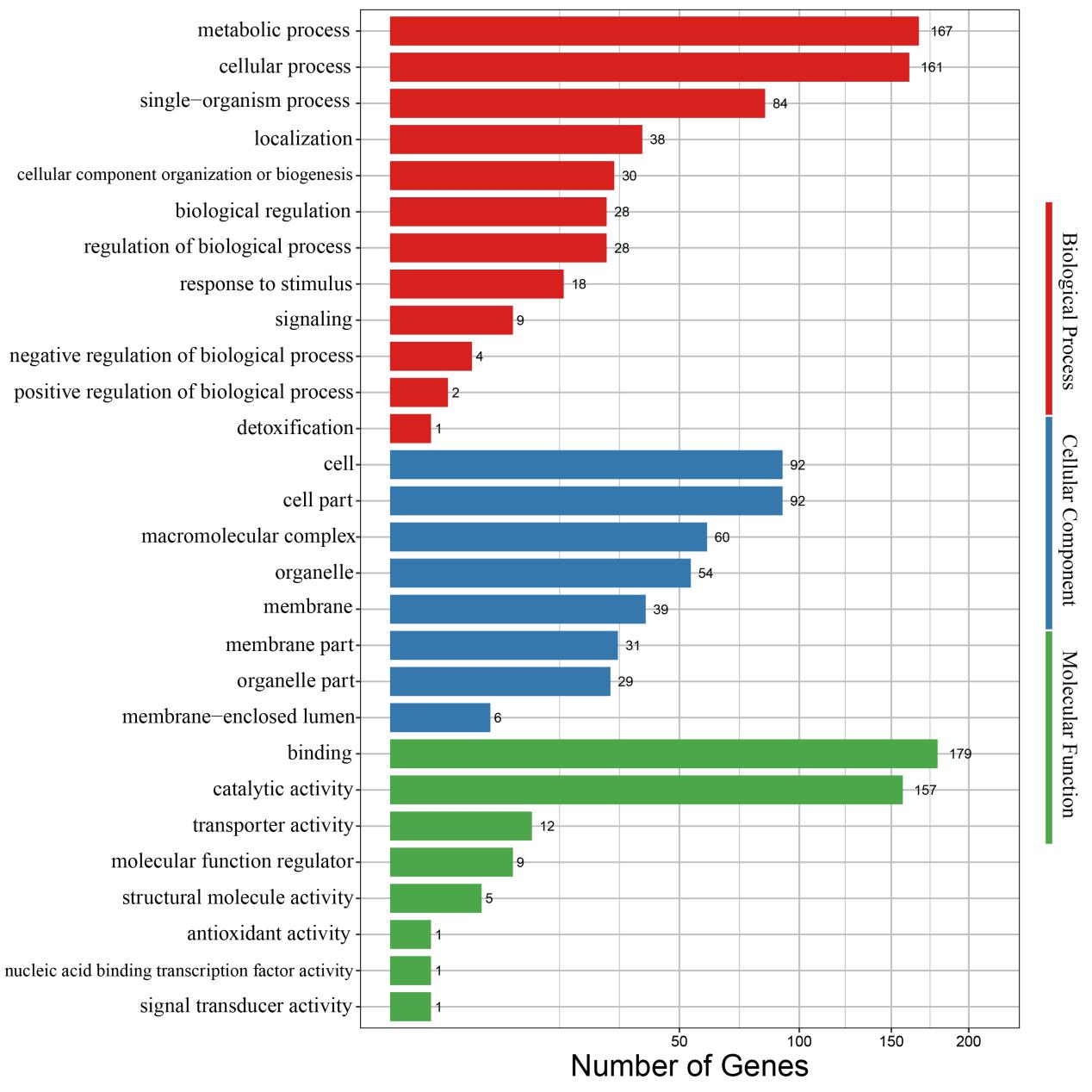


**Figure S1.** Display of GO classification results for positive selection genes

Note: The abscissa is -log10 of the enriched Qvalue value, the ordinate is GO term, the abscissa represents the number of genes in each category, and the ordinate represents the three major modules of GO classification.


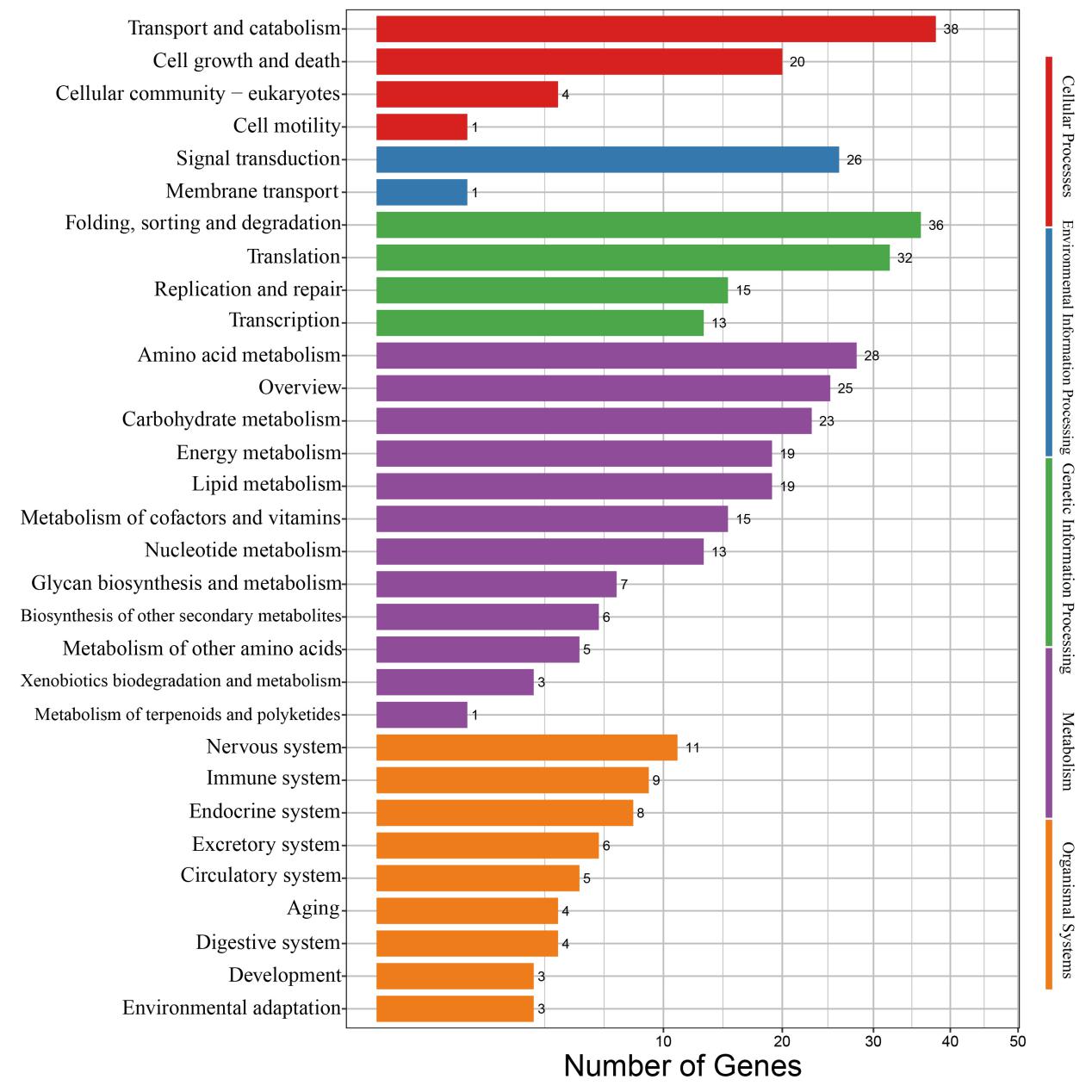


**Figure S2**. Display of the results of the positive selection gene KEGG classification

Note: The abscissa is -log10 of the enriched Qvalue value, the ordinate is KEGG term, the abscissa represents the number of genes in each category, and the ordinate represents the five major modules of KEGG classification.


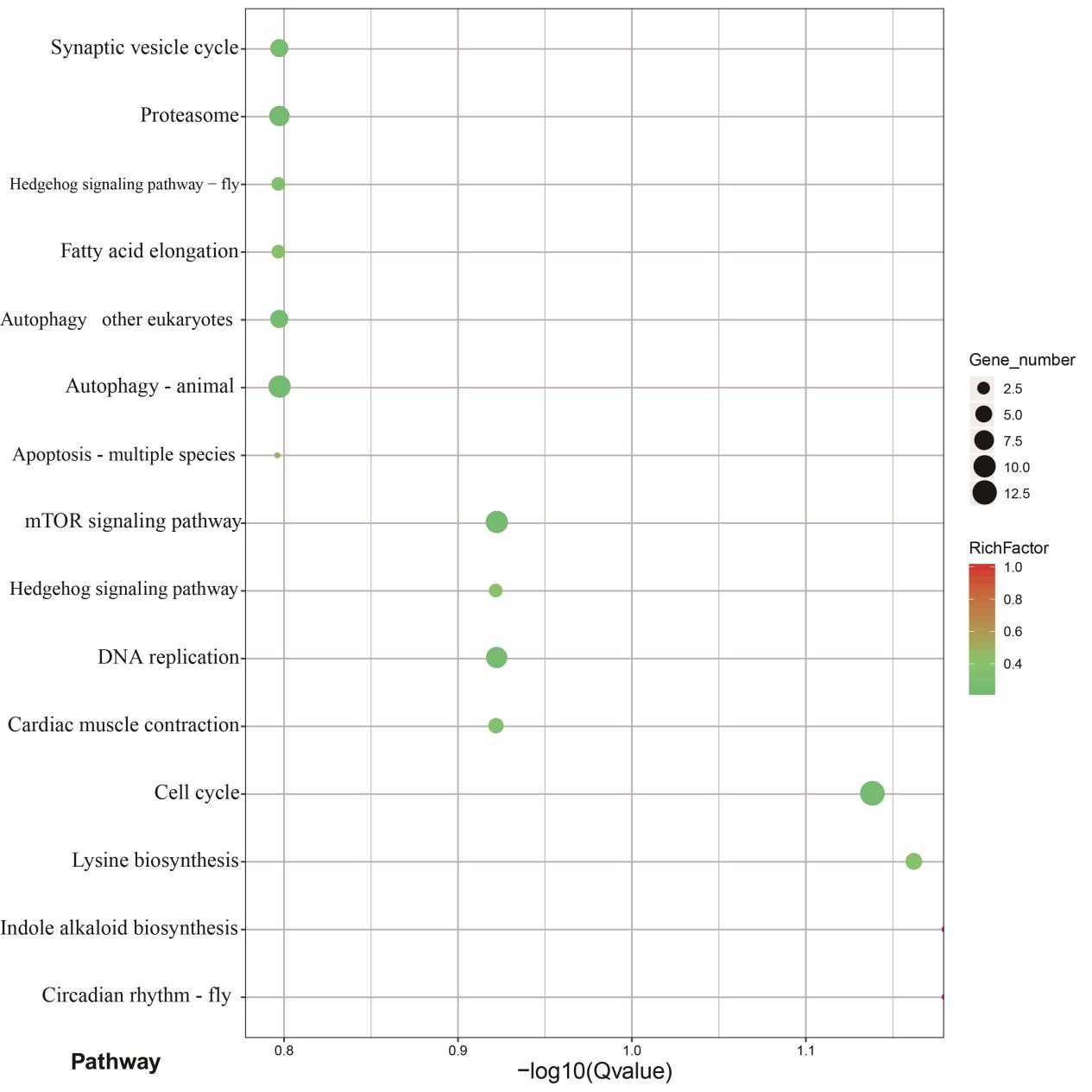


**Figure S3.** Display of KEGG enrichment results for positive selection genes

Note: The abscissa is -log10 of the enriched Qvalue value, the ordinate is KEGG term, the abscissa represents the number of genes in each category, and the ordinate represents the enriched pathway.


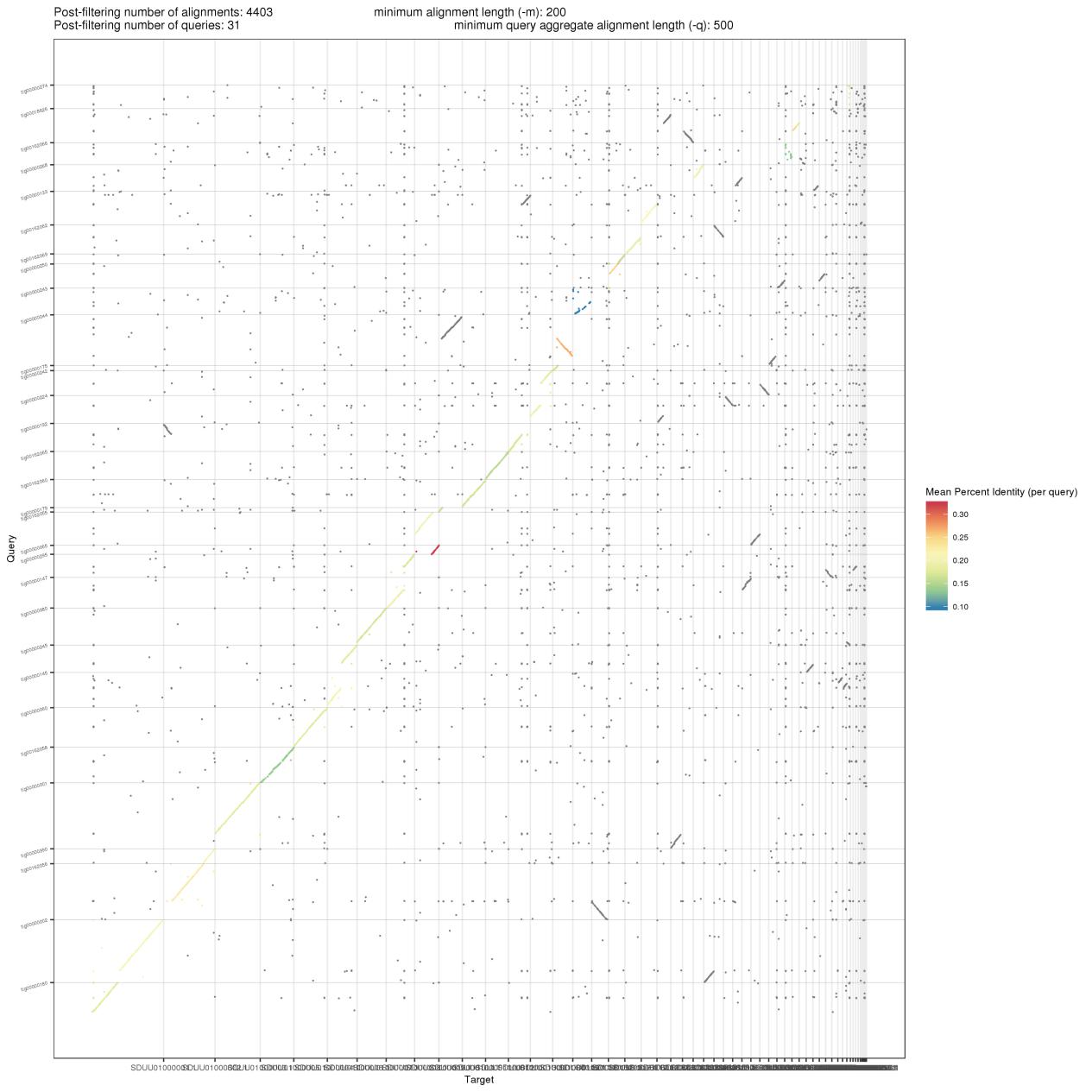


**Figure S4.** Collinearity results of *Morchella eohespera* and *Morchella sextelata*

Note: The abscissa represents *M. sextelata*, and the ordinate represents *M. eohespera*;


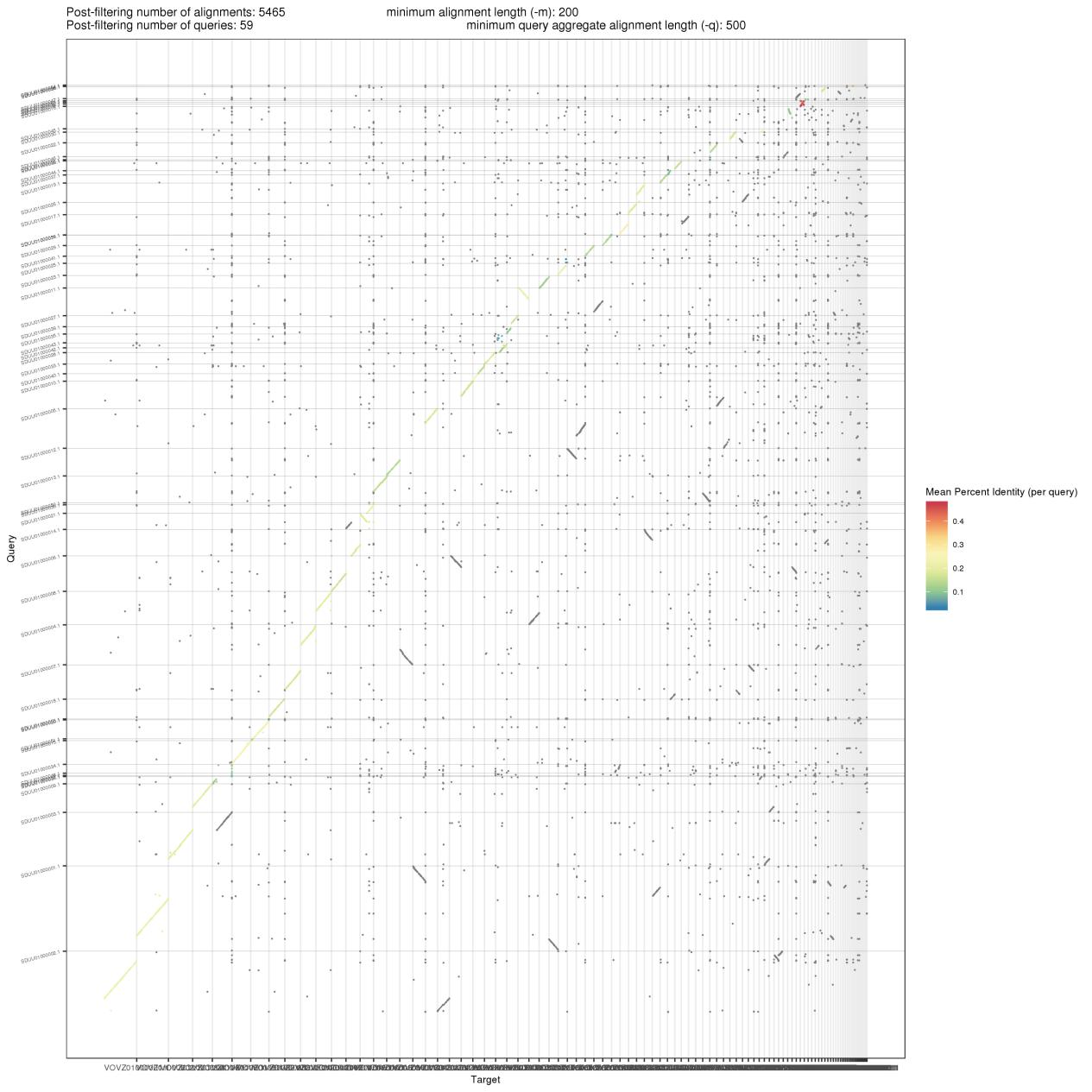


**Figure S5.** Collinearity results of *Morchella sextelata* and *Morchella conica.*

Note: The abscissa represents *M. conica*, and the ordinate represents *M. sextelata*.

**
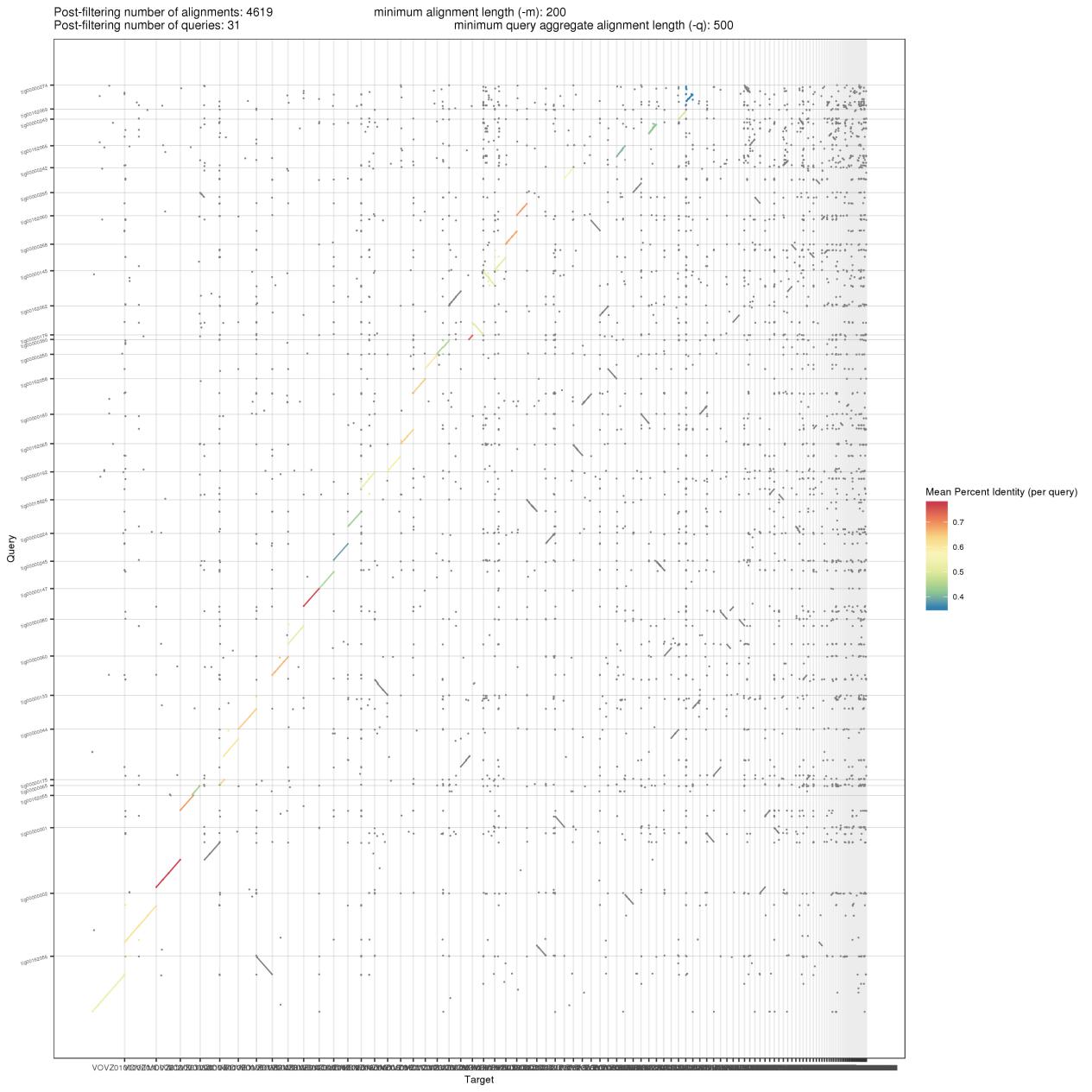
**

**Figure S6.** Collinearity results of *Morchella eohespera* and *Morchella conica*

Note: The abscissa represents *M*. *conica*, and the ordinate represents *M*. *eohespera*.


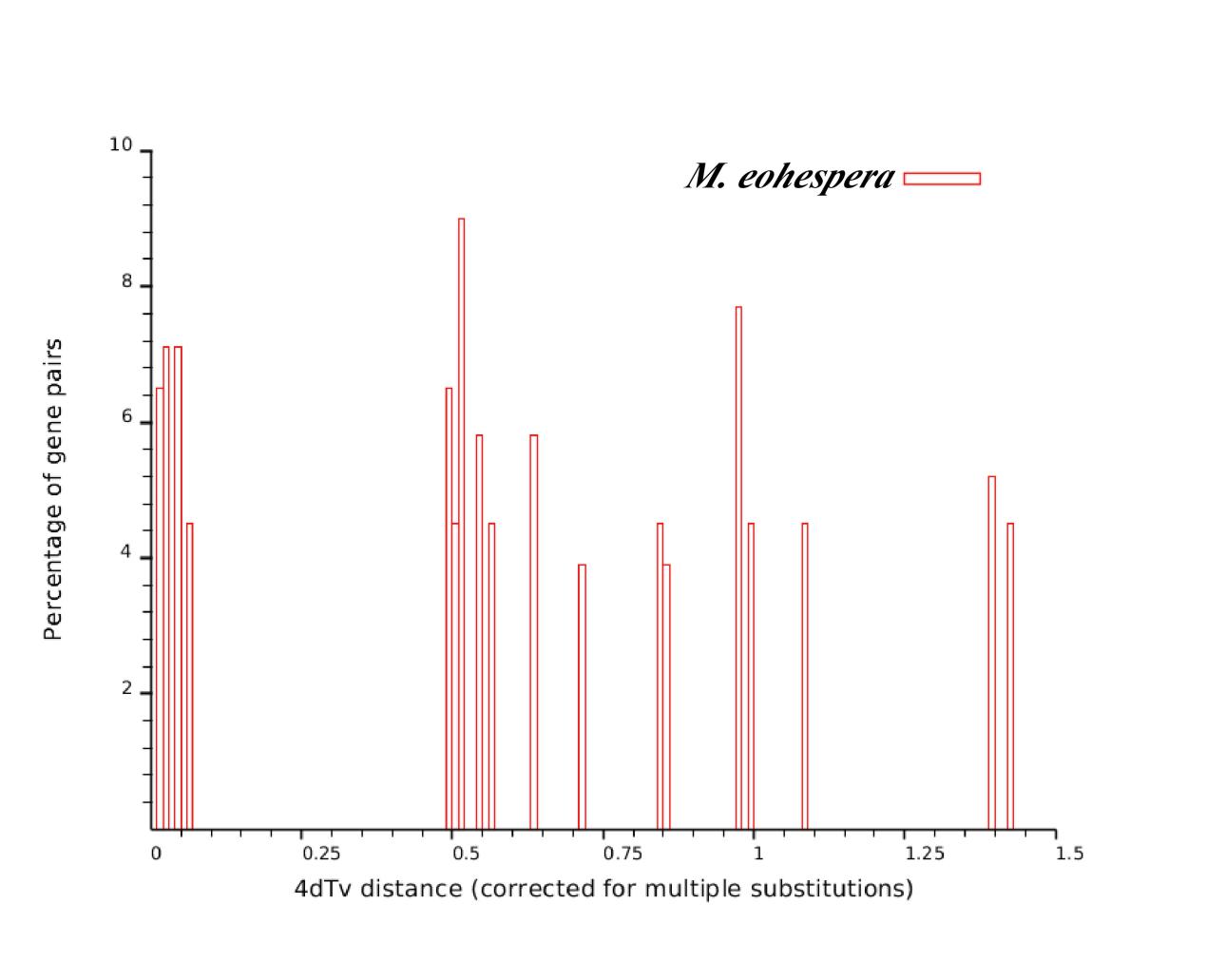


**Figure S7. 4DTV distribution map of *Morchella eohespera***

Note: The abscissa represents 4DTV, and the ordinate represents the percentage of 4DTV corresponding gene pairs.


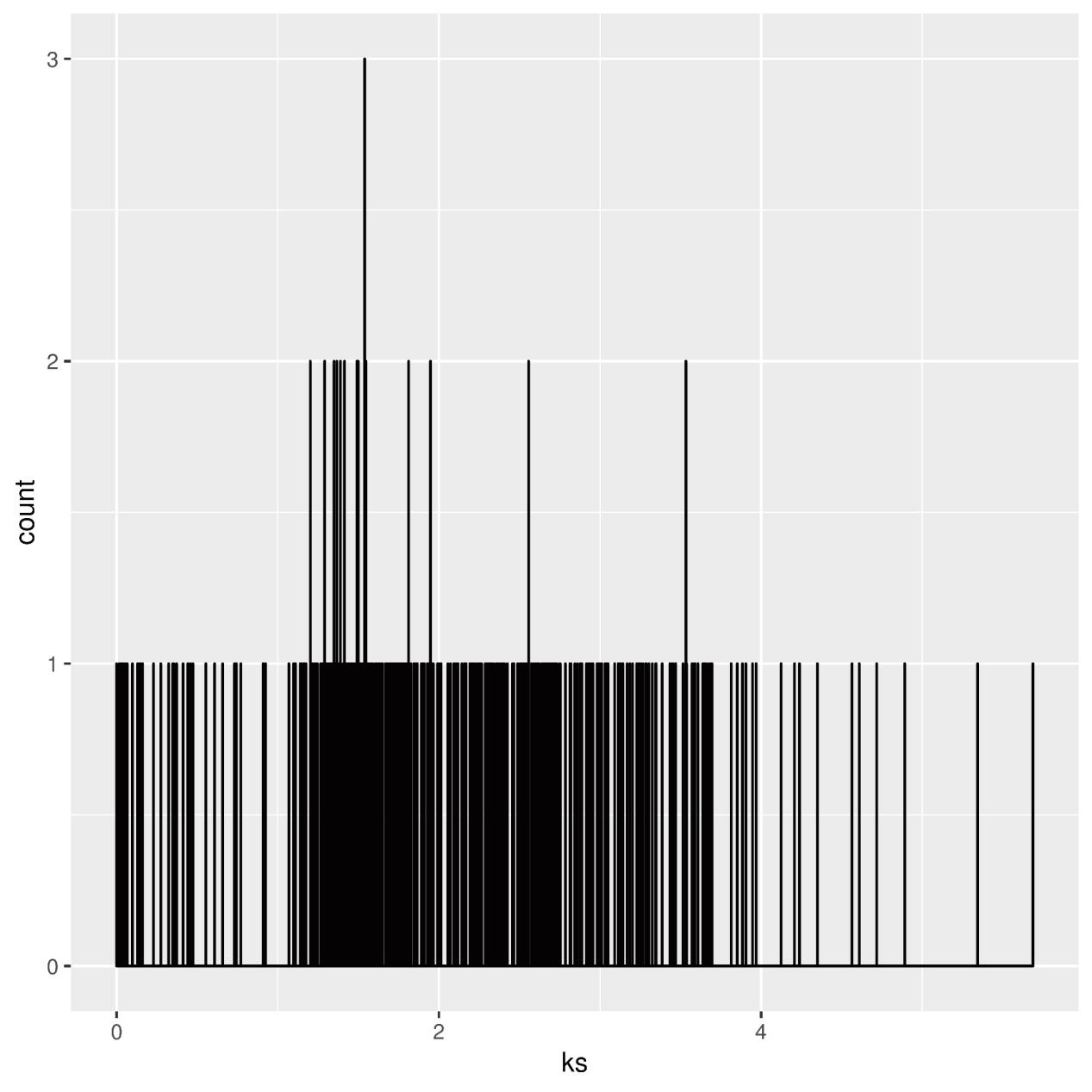


**Figure S8.** Ks distribution map of *Morchella eohespera*

Note: The abscissa represents the ks value, and the ordinate represents the number of Ks values.
